# Affordable Sinskey Hook Goniotomy and Cataract Surgery in Black and Afro-Latino Patients Diagnosed with Glaucoma: Retrospective Real-World One-Year Results

**DOI:** 10.3390/jcm14103266

**Published:** 2025-05-08

**Authors:** Idaima Calderon, Daniel Laroche

**Affiliations:** 1 Advanced Eyecare of New York, New York, NY 10027, USA; idaima.calderon@gmail.com; 2 New York Eye and Ear of Mount Sinai, New York, NY 10027, USA

**Keywords:** Sinskey hook, goniotomy, MIGS, glaucoma

## Abstract

**Background/Objectives:** This study aimed to evaluate the effectiveness of early phacoemulsification cataract surgery combined with goniotomy using a Sinskey hook in patients with glaucoma. **Methods**: This was a retrospective study conducted at Advanced Eye Care of New York; a private practice located in New York City. Most patients carried diagnoses of mild to moderate glaucoma and were mainly Black and Afro-Latino in origin. The patients included in this study were those who underwent early phacoemulsification cataract surgery combined with goniotomy performed with a reusable Sinskey hook (Ambler 200 μm tip) between January 2022 and August 2023 and completed 1 year of follow-up. The primary outcome measures were intraocular pressure, number of medications used, visual acuity, visual field indices, pre-/post-operative spherical refractive error, and adverse events. **Results**: A total of 121 eyes were identified with a 1-year follow-up that underwent this combined surgery. The mean age was 65. The mean medically treated pre-operative intraocular pressure ± standard deviation (SD) was lowered from 16.40 ± 4.5 mmHg at baseline to 14.66 ± 3.1 mmHg at 1 year, a statistically significant reduction of 10.6%. There was an 82% reduction in the mean ± SD number of intraocular pressure-lowering medications used, from 1.67 ± 1.2 at baseline to 0.30 ± 0.8 at 1 year. Out of the 121 eyes, 83% (103 eyes) remained medication-free at 1-year post-operation. Post-operatively, there were five IOP spikes (IOP ≥ 30 mmHg) and eight hyphemas that were noted, addressed, and resolved. **Conclusions**: Early cataract surgery combined with Sinskey hook goniotomy microinvasive surgery effectively reduced intraocular pressure and medication burden in this cohort of predominantly Black and Afro-Latino patients diagnosed with glaucoma with 1-year follow-up.

## 1. Introduction

Glaucoma is the leading cause of preventable blindness globally and disproportionally affects Black and Afro-Latino populations [1]. A 2024 meta-analysis estimates that glaucoma affects over 4 million people in the US and is 2–3 times more prevalent in Black adults than their White counterparts [1]. Other studies approximate the rate is closer to 3–4 times greater [2]. Increased intraocular pressure (IOP) is the primary risk factor for glaucoma and the main target in treatment [3]. Though the mean IOP in the general population is estimated to be approximately 15 mm Hg, prior studies have found mean pressures in untreated glaucomatous eyes often range from 18 mm Hg to mid-low 20 s [2,3]. The current therapeutic options to reduce IOP include medications, laser surgery, and incisional procedures. Though eye pressure-lowering medications are an effective way to reduce eye pressure and slow the progression of the disease, they can be limited in their ability to completely halt progression [4,5]. Factors such as suboptimal medication adherence (missed doses) and diminished persistence (continued use of medication over time) are associated with worsening visual field (VF) progression and are problems that disproportionately affect high-risk sociodemographic groups [4,5,6]. Health disparities like these are more likely to affect Black and Afro-Latino communities, as these populations often face greater barriers and inequities related to socioeconomic status, education, language fluency, health literacy, insurance coverage, and access to care, and are a byproduct of a greater historical context and systemic issues [6,7,8,9,10,11].

Early cataract surgery combined with trabecular bypass surgery represents a viable alternative intervention that effectively lowers intraocular pressure (IOP) and reduces reliance on medications. This approach merits consideration as a first-line strategy for patients aged 50 and above diagnosed with glaucoma- and age-related lens enlargement, particularly when performed by skilled surgeons [9]. Cataract and age-related increases in lens size and thickness can play a major role in glaucoma through mechanisms such as pupillary block and by contributing to pigment liberation through iridolenticular contact and the obstruction of the trabecular meshwork [9]. Cataract surgery has been proven to safely and effectively lower IOP by 13–71% in patients with comorbid open- or closed-angle glaucoma [9,12,13,14,15]. The EAGLE study [14] found early lens extraction to be more effective than laser iridotomy in treating angle closure glaucoma and recommended it as a first-line treatment. Early cataract surgery and goniotomy have also been shown to be efficacious in patients with open-angle glaucoma [16,17,18,19]. As glaucoma within the Black population has been shown to present at earlier ages and with more advanced disease and complications [7,8,18], early surgical intervention could help prevent vision loss and circumvent challenges related to medication adherence.

In recent years, minimally invasive glaucoma surgery (MIGS) has emerged as a widely adopted alternative for treating mild to moderate glaucoma, though the high cost of these devices remains a challenge. However, the high cost associated with using these devices [19,20] can limit their access and use in lower resource areas around the world. Several studies have attempted to shed light on more affordable MIGS options: one reported the Kahook Dual Blade as the most cost-effective device [20] and other studies have reported on more affordable surgical alternatives that use comparable techniques, such as goniotomy with a Tanito hook [19,20,21].

Previously, we reported our preliminary data using another inexpensive device to perform MIGS in conjunction with cataract surgery, the Sinskey hook [19]. We commented on the advantage of the Sinskey hook’s 200-µm tip size and smoothness, which corresponded well with the size of the trabecular meshwork and reduced the potential of bleeding from injury to the ciliary body or the back wall of Schlemm’s canal. Our preliminary findings suggested that cataract extraction paired with goniotomy using the Sinskey hook was an affordable and effective method to lower IOP and medication burden in Black and Afro-Latino patients diagnosed with glaucoma. This report aims to provide one-year surgical outcomes of patients who underwent cataract surgery with goniotomy using the Sinskey hook.

## 2. Materials and Methods

This retrospective single-center analysis examines surgical outcomes in a predominantly Black and Afro-Latino (self-identified) patient population with glaucoma who received combined phacoemulsification cataract surgery and goniotomy utilizing a 200 μm tip Sinskey hook (Ambler Surgical, Exton, PA, USA). Patients of all races who carried a diagnosis of mild to moderate open-angle or narrow-angle glaucoma and who underwent this procedure between January 2022 and August 2023 and had completed 1-year follow-up were included in this study. Patients with severe glaucoma and/or synechial angle closure were not offered this procedure and were, thus, excluded. Retrospective patient data were collected from a private practice located in Harlem, New York City, USA, and Queens Village, NY, USA. Glaucoma subtypes were noted.

Informed consent was waived due to the retrospective nature of the study, as approved by the Icahn School of Medicine of Mount Sinai Institutional Review Board (IRB). The study was conducted in accordance with the Declaration of Helsinki.

Data were obtained throughout the clinical course of each patient, at their pre-operative visit and their post-operative visits at one day, one month, six months, and one year following surgery. The goal was to significantly reduce intraocular pressure and the amount of medication needed in all the included patients, as compared to baseline. However, the medical needs, target intraocular pressure, and baseline pressure measurements were varied and specific to each individual patient. As such, the decision to restart or eliminate medication was assessed during patient follow-up visits and then performed as clinically indicated.

A reusable, straight Sinskey hook, an instrument commonly used in cataract surgery, was utilized to open the trabecular meshwork and the inner wall of Schlemm’s canal. This ophthalmic tool leaves a 200 μm wide opening and reduces bleeding risk, due to the blunt tip and smooth back end of the device. The device was reused several times without any noted difference in efficacy. If the device were to become damaged during sterilization or other handling, then a new one would be used. A total of four devices were used between four operating rooms, with one replaced due to damage to the tip during sterilization.

The study’s primary outcome measures included intraocular pressure, amount of medication used, best corrected visual acuity (BCVA) pre-/post-operatively, visual field, pre-/post-operative spherical refractive error, and adverse events. Humphrey Visual Field Analyzer (Zeiss Humphrey Systems, San Leandro, CA, USA) 24-2 results were reviewed before the surgery and at 1 year post-operation. Target post-operative refraction was between 0 to −0.5 diopters by ocular biometry.

### Procedure

**Figure 1:**
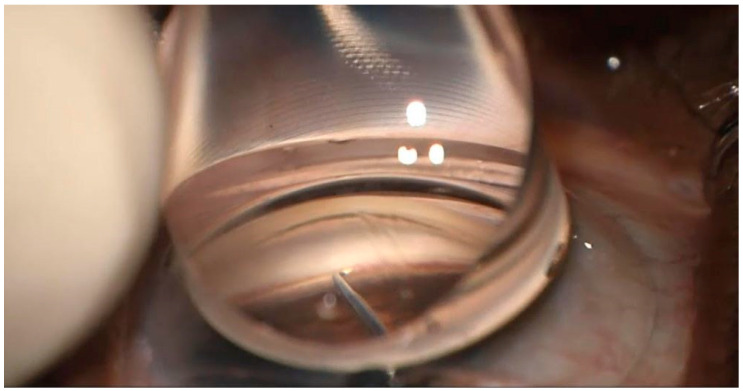
Goniotomy using a Sinskey hook.

The patients were administered a pre-operative regimen of prednisolone acetate 1% (Allergan, Dublin, Ireland) four times daily (QID), ofloxacin (Rising, Saddle Brook, NJ, USA) QID, and Ketorolac 0.4% (Allergan, Dublin, Ireland) three times daily (TID), starting 3 days prior to surgery. Preparations for the eye on the day of surgery included betadine and draping, and the application of topical anesthesia. Phacoemulsification cataract surgery was performed through a clear cornea, and an intraocular lens was implanted. To deepen the angle, EndoCoat (Abbott, Chicago, IL, USA) was injected into the anterior chamber and on the cornea. To optimize visualization, adjustments were then made to tilt the patient’s head to approximately 45° away from the surgeon, while the microscope was tilted toward the surgeon at the same angle. Structures in the nasal angle were visualized after focusing the microscope through a direct gonio lens (Katena, Troy Hills, NJ, USA) that was positioned on top of the eye. A Microvitreoretinal (MVR) blade was then utilized to penetrate the trabecular meshwork and create an opening in Schlemm’s canal. Following this, a Sinskey hook was introduced into Schlemm’s canal and passed approximately 2–3 clock hours to the left and then to the right to unroof the canal (Figure 1). After completing this step, the Sinskey hook was withdrawn and balanced salt solution was injected via paracentesis, and the wound was hydrated and closed pressurizing the eye to about 20 mmHG. At the conclusion of the procedure, an intracameral injection of diluted Vigamox (Alcon, Geneva, Switzerland) mixed in a 50/50 ratio with a balanced saline solution was administered via paracentesis. It was observed that in all the patients, the nasal angle remained open following cataract surgery. The corneal incisions were hydrated and sealed effectively to ensure no leakage. Post-operatively, the patients were immediately placed in a seated position to keep their heads elevated above waist level to reduce episcleral venous pressure.

The patients received instructions to sleep in an elevated position for four nights post-operatively to facilitate the settling and clearing of any potential heme reflux that could cause visual obstruction. They were advised to continue using ofloxacin QID for seven days. Prednisolone acetate 1% was tapered over four weeks (QID to TID to BID to daily—each dose taken for seven days) before discontinuation. Ketorolac 0.4% was continued TID for four weeks before being discontinued. The patients were also instructed to discontinue all glaucoma medications in the operated eye immediately after surgery. This comprehensive protocol ensured proper healing while optimizing surgical outcomes and maintaining intraocular pressure control.

## 3. Results

Of the participants who underwent phacoemulsification cataract extraction combined with a Sinskey hook goniotomy procedure and completed a 1-year follow-up, there were 121 eyes identified and enrolled in the study. The mean age of the participants was 65 ± 10 years, and 70% were female and 30% were male (Table 1). The self-described race and ethnicity demographics of our study population were 87% Black or Latino, 4% Non-Hispanic White, and 9% Asian (Table 2). Fifty (41%) participants were diagnosed with primary open-angle glaucoma, twenty-eight (23%) with ocular hypertension, twenty-four (20%) with angle-closure glaucoma, nine (7%) were glaucoma suspects, seven (6%) had narrow angles, and three (2%) had pigmentary glaucoma. All the baseline characteristics and calculations were collected on medically treated eyes, and included IOP, number of ocular hypertensive medications used, BCVA, and visual field index (VFI) and mean deviation (MD) on visual field test (VFT) (Table 1).

A statistically significant decrease in mean IOP for all the eyes was achieved following the phacoemulsification cataract surgery with Sinskey hook goniotomy at one-year follow-up (Table 3). The baseline mean pre-operative IOP and standard deviation for all the eyes was 16.40 ± 4.5 mmHg. As our study did not conduct a pre-operative washout of medications, our population started at a relatively lower baseline IOP. Nevertheless, on post-operative day 1, the mean IOP and standard deviation for all eyes were significantly lowered to 14.31 ± 6.2. This statistically significant reduction in IOP remained at one year, with the mean IOP and standard deviation for all the eyes at 14.66 ± 3.1 mmHg. This is a 10.6% reduction from baseline.

At baseline, 86% (104 out of 121 eyes) were taking intraocular pressure-lowering medications. The mean ± standard deviation number of pressure-lowering medications used for all the eyes at baseline was 1.67 ± 1.2. At 1-year, we observed a statistically significant decrease to 0.30 ± 0.8, an 82% reduction. Out of the 121 eyes, 83% (103 eyes) remained medication-free at 1 year post-operation.

There was an improvement in the visual acuity and visual field measurements in mean totals from the baseline to the one-year follow-up. The mean BCVA (logMAR) and standard deviation in all the eyes improved from 0.37 ± 0.3 at baseline to 0.12 ± 0.3 at one year post-operation. There was a slight decrease in the mean spherical equivalent in all the eyes (Table 4) due to several outliers in the baseline data, which is reflected in the wider standard deviation. The mean pre-operative VFI% and MD in all the eyes were 86.2% and −6.63, respectively. The mean post-operative VFI% and MD in all the eyes were 89.0% and −5.48, respectively. Post-operatively, there were five IOP spikes (IOP ≥ 30 mmHg) that were treated and eight hyphemas that were noted, addressed, and resolved. All the complications were treated in the usual manner and resolved within a few days to two weeks.

## 4. Discussion

This study shows that our surgical approach of early cataract surgery combined with Sinskey hook goniotomy was effective in improving visual acuity, reducing intraocular pressure, and decreasing medication burden one year following surgery. We found that our technique demonstrated a relatively favorable safety profile, with only a few minor adverse effects, all of which resolved without sequelae.

Cataract surgery paired with goniotomy should be considered at earlier stages of mild-to-moderate glaucoma and as an initial intervention for patients aged 50+ with glaucoma related to lens enlargement. Though IOP-lowering drops are effective in slowing disease progression, their effects are limited by the patient’s ability to adhere to the medication regimen consistently over time. With studies estimating that at least 50% of the patients in the US are missing doses [4], it is important to explore alternative treatment options that can provide stable IOP reduction without facing the same vulnerabilities to external variables (such as medication costs, side effects, and confusion about regimen). The recent focus on MIGS provides a path for just that, with many studies demonstrating these new approaches and devices to be effective in the reduction in IOP and in the management of glaucoma, especially when paired with cataract surgery [13,22,23,24].

Consistent with this growing body of literature, our surgical approach was able to achieve and sustain similar levels of IOP control as other MIGS devices. One recent systematic review analyzed the clinical outcomes of MIGS devices with and without cataract surgery in 74 studies and reported that mean IOP at 12 months ranged between 11.4 and 18.1 mmHg [24], which is aligned with our result of 14.7 mmHg. Our results are also consistent with several studies that reported the 12-month outcomes of the Kahook Dual Blade with cataract surgery and showed mean IOPs ranging between 12.4 and 15.4 mmHg [25,26,27]. Although the 10.6% IOP reduction in our study is modest, this statistically significant reduction also holds clinical significance in that the lower IOP and decreased diurnal fluctuation reduce the risk of glaucoma progression in this population. Additionally, recent clinical trials have demonstrated that these reductions in IOP with MIGS can be sustainable in even longer terms. The largest prospective randomized clinical trial in MIGS, the HORIZON trial, has published the results for the Hydrus Microstent showing that IOP reduction was improved with combined surgery and maintained over 5 years, with many not requiring additional pharmacological therapy [22].

Similarly, the outcomes of our study demonstrated that combined cataract surgery with Sinskey hook goniotomy reduced medication burden by 82%, with 83% of the patients remaining medication free at one year. Glaucoma medication adherence and persistence rates in the US are suboptimal and can be influenced by factors such as medication costs, side effects, limited health literacy, access to care, and interference with quality of life, among many others [4,5,28]. Since lower adherence rates are associated with increased visual field progression [4,5], offering an early surgical treatment alternative in populations that are disproportionately affected by these challenges could help address some of these disparities, reduce disease burden, and help preserve vision. Our findings reinforce this argument and align with the other MIGS literature [24], in that we observed no deterioration in the post-operative visual field of our patients, suggesting a reduction in visual field progression.

One of the frequently reported advantages of MIGS devices is that they generally have better safety profiles than trabeculectomy and require less invasive surgery [13,22,23,24]. The added benefit of performing a goniotomy with the Sinskey hook is that it does not carry the same potential risk of complications that can occur with permanently implanted MIGS devices, such as malposition, migration, and obstruction [29]. Furthermore, the smooth tip of the Sinskey hook protects the back wall of Schlemm’s canal and the 200 μm width fits nicely within the canal, allowing one to open it bluntly and in a circumferential manner. Only four clock hours are needed to open the canal to obtain efficacy and reduce complications of bleeding that can occur from sharper devices, such as a bent cystotome or needle goniotomy.

Further distinguishing the Sinskey hook is that it is a more affordable option that offers results comparable to other excisional goniotomy devices and MIGS. In a study conducted by Sood and Chen [20], a cost analysis was performed comparing the affordability of different MIGS in relation to their efficacy in reducing IOP (cost per mmHg of intraocular pressure reduction). The devices all performed similarly in efficacy; however, they found that cost-effectiveness was best in the Kahook Dual Blade, followed by the Hydrus Microstent, Trabectome, and then iStent Inject [20]. The Sinskey hook has shown similar results in regard to efficacy, but is even less expensive than the Kahook Dual Blade and Tanito hook. Since the direct costs of glaucoma care correlate strongly with disease severity, the Sinskey hook provides a cost-effective treatment option [30].

As an affordable alternative to other microinvasive surgical techniques, this approach carries important implications for patients in resource-poor areas in that it reduces barriers to care and increases accessibility to glaucoma surgical treatment. Low-resource regions of the world with a significant glaucoma burden, such as sub-Saharan Africa, Southeast Asia, and the Caribbean, often face increased rates of blindness from glaucoma [31,32]. This can be due to factors such as lack of access to care, challenges with medication adherence, and costs of treatment [31,32]. A recent cross-sectional study collected data from 10 sub-Saharan African countries and found that the most common reasons for patients to decline surgical intervention were fear and the cost of treatment [32]. The Sinskey hook is easily accessible, often coming in cataract surgical sets, and its reusability also makes it an ideal surgical alternative that could lower financial barriers to treatment for these patients. With its enhanced safety profile, this procedure provides hesitant patients an alternative means to avoid more invasive interventions, such as trabeculectomy.

Comparable outcomes may be observed with earlier manual small-incision cataract surgery (MSICS), where lens removal is performed in glaucoma patients. A study conducted in the Congo reported a 37.39% reduction in intraocular pressure (IOP) following MSICS in glaucomatous patients, with pre-operative IOP decreasing from 23.16 ± 5.68 mmHg to 14.5 ± 2.7 mmHg post-operatively. Further research should assess whether early MSICS alone or combined with Sinskey hook goniotomy offers greater efficacy for mild to moderate glaucoma cases [33]. Offering earlier, safer, more affordable surgery that reduces the need for long-term topical treatment would help address many of the barriers mentioned in this article and decrease the risk of disease progression. This should be performed by experienced surgeons. According to the 2023 data from the Association of American Medical Colleges (AAMC), there are approximately 19,000 ophthalmologists practicing in the US [34]. Of those, only about 10,000 ophthalmologists perform cataract surgery, with even fewer performing cataract surgery and goniotomy [35]. The surgeon in this report has over 25 years of experience in cataract and glaucoma surgery and in preventing and managing complications to ensure optimal outcomes. We need to train more surgeons to be highly skilled with uncomplicated surgery to ensure excellent outcomes globally.

This study is limited by its retrospective design and absence of a control group. This makes it challenging to infer conclusions of causality between our procedure and the observed outcomes. Though these results are promising and show consistency with those demonstrated in larger clinical trials of other MIGS, utilizing a prospective design with a comparison group as a control arm (e.g., standard cataract surgery alone or other MIGS techniques) in future studies would strengthen the internal validity. Though we did not measure lens thickness in this study, future interventional studies should incorporate this variable to gain a deeper understanding of how lens removal and MIGS can contribute to IOP reduction in different types of glaucoma.

## 5. Conclusions

Early cataract surgery combined with goniotomy using a Sinskey hook serves as an effective microinvasive surgical alternative, demonstrating reduced intraocular pressure and decreased reliance on ocular hypertensive medications in a cohort primarily composed of Black and Afro-Latino patients with glaucoma, with the outcomes tracked over a 1-year follow-up period. Further research and increased levels of participation from patients of diverse backgrounds are needed to gain a true assessment of the safety and efficacy of glaucoma treatments and interventions in these populations. Longer-term prospective studies that assess the clinical outcomes and economic impact of this combined surgery are necessary to have a better representation of its effects on this chronic, life-long disease.

## Figures and Tables

**Table 1:** Baseline characteristics of participants.

Variable	Category	Statistics, n Total = 121
Age (years)	Mean ± SD	65 ± 10.4
Gender, n (%)	Female	85 (71%)
	Male	36 (30%)
Eye, n (%)	Right	66 (55%)
	Left	55 (45%)
Baseline IOP (mmHg)	Mean ± SD	16.40 ± 4.5
Ocular hypertensive medications	Mean ± SD	1.67 ± 1.2
Number of ocular hypertensive medications used, (%)	0	17 (14%)
	1	57 (47%)
	2	8 (7%)
	3	27 (22%)
	≥4	12 (10%)
Visual acuity (logMar)	Mean ± SD	0.37 ± 0.3
MD on VFT	Mean ± SD	−6.63 ± 7.0
**VFI on VFT**	Mean ± SD	86.2% ± 20.2

**Table 2:** Race and ethnic origin of participants.

	N Total = 121
**Black, n (%)**	103 (85%)
**White, n (%)**	7 (6%)
**Asian, n (%)**	11 (9%)
**Hispanic, total, n (%)**	15 (12%)
**Hispanic, Black, n (%)**	13 (11%)
**Hispanic, White, n (%)**	2 (2%)
**Non-Hispanic, White only, n (%)**	5 (4%)

**Table 3:** Post-operative data for all patients who underwent phacoemulsification cataract surgery with Sinskey hook goniotomy.

Timepoint	Intraocular Pressure (IOP) mmHG, Mean ± SD	Ocular Hypertensive Medications, Mean ± SD	BCVA logMAR, Mean ± SD	Visual Field Test, Mean Deviation ± SD
Baseline	16.40 ± 4.5	1.67 ± 1.2	0.37 ± 0.3	−6.63 ± 7.0
Post-operative day 1	14.31 * ± 6.2	0 *	-	
1 month	15.96 ± 4.8	0.18 * ± 0.7	0.13 ± 0.3	
3 months	13.87 * ± 4.4	0.27 * ± 0.8	0.11 ± 0.2	
6 months	14.27 * ± 3.2	0.30 * ± 0.8	0.11 ± 0.3	
1 year	14.66 * ± 3.1	0.30 * ± 0.8	0.12 ± 0.3	−5.48 ± 6.1

* Statistically significant reduction from baseline.

**Table 4:** Mean spherical equivalents (SEs) for all eyes included.

	Mean Spherical Equivalent ± SD
Pre-operative SE for all eyes	−0.19 ± 3.51
Post-operative SE for all eyes	−0.53 ± 0.90

## Data Availability

The data presented in this study are available upon request from the corresponding author due to the privacy of patient information.
